# SilverSil: A New Class of Antibacterial Materials of Broad Scope

**DOI:** 10.1002/open.202000016

**Published:** 2020-04-09

**Authors:** Keren Trabelsi, Rosaria Ciriminna, Yael Albo, Mario Pagliaro

**Affiliations:** ^1^ Department Chemical Engineering, Materials and Biotechnology The Center for Radical Reactions Ariel University Jerusalem Israel; ^2^ Istituto per lo Studio dei Materiali Nanostrutturati, CNR via U. La Malfa 153 90146 Palermo Italy

**Keywords:** Antibacterial activity, Silver, *Staphylococcus aureus*, *Escherichia coli*, ORMOSIL

## Abstract

Consisting of organically modified silica (ORMOSIL) physically doped with Ag nanoparticles, the SilverSil new class of antibacterial materials of broad scope reported herein shows remarkably high and stable activity against representative Gram‐positive and Gram‐negative bacteria. The low cost, ease of application and excellent health and environmental profile of SilverSil hybrid glassy coatings open the route to their widespread utilization across domestic, hospital, school, industrial and commercial environments and in consumer products.

## Graphical abstract

**Broad scope solid antibacterial? Try SilverSil** ORMOSIL coatings physically doped with Ag nanoparticles show high and stable activity against Gram‐positive and Gram‐negative bacteria. The low cost, ease of preparation and the excellent health and environmental profile of hybrid glassy coatings open the route to their utilization across different environments and different consumer products.

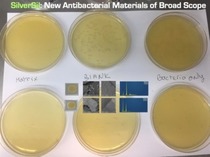

Driven by the increasing resistance to conventional antibacterial (AB) chemotherapy of many pathogens including meticillin‐resistant *Staphylococcus aureus*,^1^ the search of new broad scope antibacterial agents is the aim of intense research activities carried out in numerous countries.^2^ One approach focuses on the antimicrobial activity of plant extracts and phytochemicals, often in combination with antibiotics.^3^ Another makes use of inorganic species such as silver whose toxicity to human cells is much lower than to bacteria.^4^ Known and used in the medical field since ancient times, the antibacterial activity of silver ions (Ag^+^) and silver nanoparticles (NPs) is being increasingly understood.^5^ Silver acts in multiple ways on bacteria: Ag^+^ and Ag NP enter the cell membrane by porin proteins causing cell membrane rupture, pore formation and cytoplasmic leakage.^5^ Within the cell membrane, the metal drives the formation of several highly oxidizing species such as hydroxyl and superoxide free radicals, and H_2_O_2_ which quickly oxidise DNA and denaturate proteins.^6^ Recently, Avnir and co‐workers demonstrated how Ag^+^ species are not deactivated by the killing mechanism, with the dead bacteria serving as an efficient sustained release reservoir for releasing the lethal metallic cations for further action against other live bacteria.^7^

Starting in the early 2000s several companies across the world started to embed Ag nanoparticles in antibacterial consumer goods including undergarments, T‐shirts, towels, shirts and socks, but also in washing machines, soft toys and even personal care products such as toothpaste.^8^ Washing fabrics functionalized with Ag NPs releases silver, but this does not affect the antimicrobial efficacy of the washed fabrics as shown for example by >99.9 % inhibition of *Escherichia coli* growth on textiles retaining as little as 2 ppb Ag after prolonged washing.^9^ Knowledge of the impact of silver nanoparticles on human health and on the environment is still limited, especially concerning possible long‐term toxic effects. For example, recently scholars in Taiwan reported the first evidence of both cytotoxicity and immunotoxicity of Ag NPs on the leukocytes of common bottlenose dolphins.^10^ Furthermore, it is already known that more than 90 percent of silver nanoparticles released in wastewater end up in the nutrient‐rich biosolids residual of sewage treatment.^11^ These solids are often used on land as fertilizers, with plants taking up silver from soil.^12^ Silver is thus concentrated and introduced into the food chain. To prevent environmental contamination with silver nanoparticles, scholars in North America lately suggested its direct treatment in the washing machine by removing it from wastewater using an ion‐exchange resin cartridge.^13^

An alternative and even more desirable approach, since it replaces pollution control with pollution prevention, is based on improving the microencapsulation technology so that microencapsulated nanoparticles can be used to produce leach‐proof coatings with which to functionalize consumer goods. Surprisingly, the sol‐gel encapsulation of Ag NPs has received little attention.^14^ In 2015, scholars in Hungary reported the antibacterial activity of Ag^+^ ions and Ag^0^ nanoparticles sol‐gel entrapped in TiO_2_ against *Escherichia coli*. No visible‐light induced photocatalytic activity of the titania coatings was detected upon silver addition, whereas the significant original antibacterial activity was partly retained after 20 h in contact with buffered solution only for samples prepared by impregnating the porous titania coatings with 1 M AgNO_3_.^15^

More recently, the first example of antifouling coatings based on silver nanoparticles embedded in an organosilica coating obtained from tetraethylorthosilicate (TEOS) and triethyl(octyl)silane was reported.^16^ The resulting coating showed good inhibition of biocorrosion of an aluminum alloy promoted by chloride media inoculated with *Pseudomonas aeruginosa*.^16^ In general, there is a widespread need to develop new, low cost and broad scope silver‐based antibacterials avoiding the unwanted health and environmental collateral effects of silver nanoparticles, but still showing the exceptionally broad scope and drug‐resistant activity of nanoparticulate silver against human pathogens, including *S. aureus*.

Now, we report the discovery that SilverSil, a new class of antibacterial materials comprised of organically modified silica (ORMOSIL) physically doped with Ag nanoparticles, shows such remarkably high and stable activity against representative Gram‐positive and Gram‐negative bacteria.

In detail, SilverSil materials of different composition (Table 1) were obtained by sol‐gel hydrolytic co‐polycondensation of TEOS and methyltriethoxysilane (MTEOS) in the presence of dissolved silver nitrate. In a typical preparation of the 70 : 30 SilverSil (70 % TEOS and 30 % MTEOS, in molar terms), 6.0 mL of aqueous HNO_3_ (0.2 M) was added dropwise to a solution consisting of TEOS (8.8 mL), MTEOS (3.4 mL) and ethanol (13.3 mL). After stirring for 10 min, the mixture was added with 125 μl of (3‐aminopropyl)triethoxysilane. An aliquot (5.0 mL) of a 6.36 mM aqueous solution of AgNO_3_ was then added dropwise, after which water (5.0 mL) and aqueous ammonia (0.01 M, 5.0 mL) were added to promote the hydrolytic polycondensation. A wet gel was quickly obtained which was dried at room temperature for approximately two weeks until a constant weight of the dry matrix was obtained.

**Table 1:** Composition of the different SilverSil materials.

SilverSil	TEOS [mol %]	MTEOS [mol %]	Ag(0) load [mmol Ag]	Specific Surface area [m^2^/g]	Specific pore volume [cm^3^/g]
90 : 10 Ag	90	10	0.0325 (0.1 % load)	394.036	0.9811
70 : 30 Ag	70	30	0.0325 (0.1 % load)	582.475	0.9918
50 : 50 Ag	50	50	0.0325 (0.1 % load)	506.046	0.9907
30 : 70 Ag	30	70	0.0325 (0.1 % load)	308.583	1.581
70 : 30 5 Ag	70	30	0.1625 (0.5 % load)	–	–
70 : 30 10 Ag	70	30	0.325 (1 % load)	–	–

The dry matrix in a mortar was crushed into a powder with a pestle, after which the dried solid was treated with aqueous NaBH_4_ (0.03 M, 100 mL) to promote reduction of the entrapped Ag^+^ ions. The material obtained was air‐dried again. The resulting xerogel was crushed in a mortar and the resulting powder used as such in antibacterial trials.

The cryogenic nitrogen‐adsorption isotherms (not shown) show that the SilverSil xerogels are mesoporous, amorphous ORMOSIL glasses of large surface area and pore specific volume (Table 1). The presence of the Ag(0) nanocrystals was confirmed by the X‐ray powder diffraction (Figure 1) using a X'pert PRO of PANalytical diffractometer, Cu−Kα X‐rays of wavelength λ=1.54056 Å with data taken in the 10°–135° 2*θ* range with a step of 0.02°.

**Figure 1:**
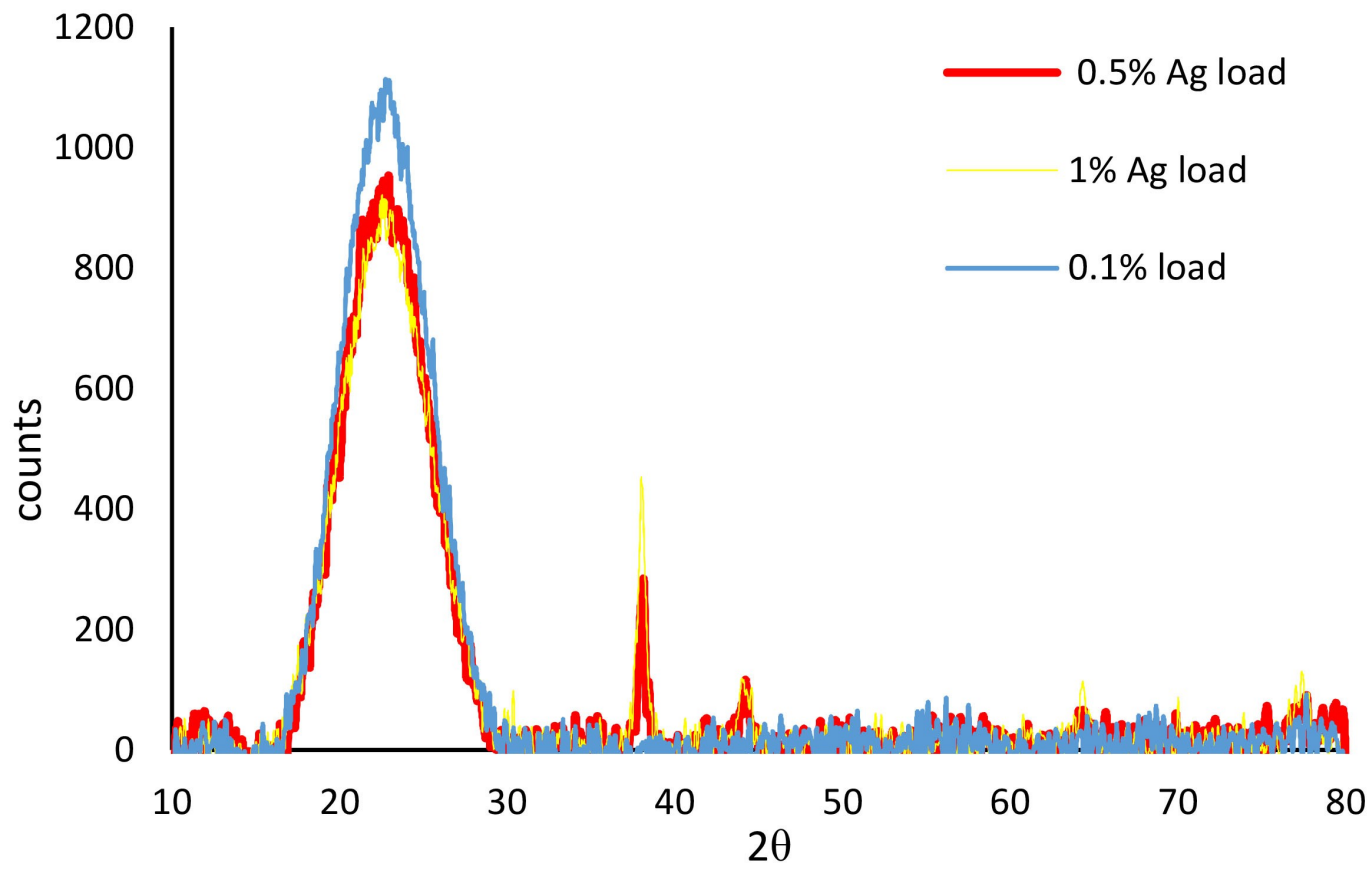
XRD patterns of 70 : 30 SilverSil with different silver loading (1 %, 0.5 % and 0.1 %).

Figure 1 shows the peaks at about 38.1°, 44.3°, corresponding to (111) and (200) planes of crystalline Ag for both 0.5 % and 1 % Ag loaded xerogels. These peaks, however, were not observed for the 70 : 30 SilverSil matrix with 0.1 % load due to the low concentration of Ag^0^ NPs.

The elements present on the surface of the xerogels, and their oxidation states, were examined by X‐ray photoelectron spectroscopy (XPS) using a Thermo Fisher Scientific, NEXSA XPS system with monochromatized Al Kα source (400micron diameter). The selected Pass Energy (“resolution”) of 200 eV for survey scans revealed the general surface composition profile, whereas 50 eV high‐resolution scans were used for quantitative analysis.

The full‐scan XPS spectrum of 70 : 30 SilverSil (Figure 2) shows the presence of Si, O and C. The binding energy for the C 1s peak at 286.1 eV was used as the reference for calibration. The XPS spectra of oxygen (O 1s) at 534.1 eV and silicon (Si 2p) at 105.1 eV originate from the organosilica matrices. The weak peak obtained by focused scan at 368.9 eV (Figure 2 inset) due to silver (Ag 3d) confirms the zero‐valent metallic nature of Ag present in SilverSil.

**Figure 2:**
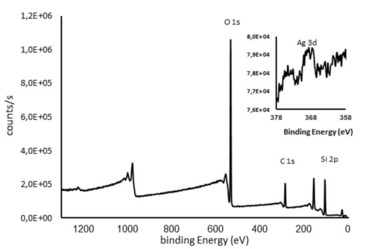
XPS spectrum of 70 : 30 SilverSil xerogel; inset: Focused scan for Ag peak.

The very weak intensity of the peak indicates that most Ag nanoparticles are located within the inner porosity of the sol‐gel matrix rather than on the xerogel surface, a well‐known and useful feature of ORMOSILs doped with metal nanoparticles^17^ or with molecular species.^18^

The energy dispersive X‐ray spectroscopy (EDX or EDS) chemical microanalysis in conjunction with scanning electron microscopy carried out with an Ultra‐High‐Resolution Maia 3 FE‐SEM using an OXFORD EDS system equipped with a X‐Max 80 detector shows that SilverSil composition includes only Si, C, O and Ag atoms.

The presence of the Ag nanocrystals in 90 : 10 and 70 : 30 SilverSil xerogels is revealed as bright spots in the SEM photographs (Figures 3a and 3d). The antimicrobial activity of the SilverSil xerogels was evaluated by testing the materials against *Staphylococcus aureus*, representative of Gram‐positive bacteria, and *Escherichia coli*, representative of Gram‐negative bacteria.

**Figure 3:**
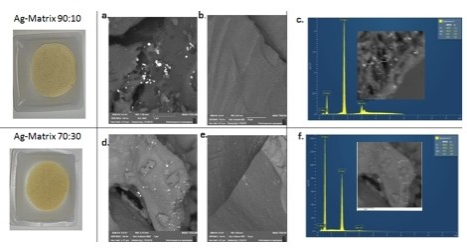
SEM images and EDX spectra of 90 : 10 SilverSil (a, b and c, respectively), and of 70 : 30 SilverSil (d, e, and f, respectively).

The general procedure for medium culture preparation was as follows. Medium culture *S. aureus* from a stock culture was inoculated into brain heart infusion broth (a growth medium for microorganisms) until 0.3 optical density (OD) was obtained (measured with Thermo Genesis 10uv, UV‐vis spectrophotometer at 600 nm).

The bacteria culture was then incubated at 37 °C for 2 hours. Medium culture *E. coli* was grown following the same procedure used for *S. aureus* growth except that the growth medium was a Luria broth medium. Bacteria were grown until obtaining OD=0.3, (log phase of growth). By then, the bacterial inoculum was centrifuged at 9,000 rpm for 10 minutes, washed with buffer phosphate (pH 7, 0.1 M) and centrifuged again. The inoculum was washed 2 times with fresh buffer. All antibacterial activity experiments were performed in water buffered at pH 7 using the aforementioned phosphate buffer (0.1 M). The inoculum was diluted to ∼10^7^ CFU/mL bacterial concentration and then diluted to get final concentration of ∼10^3^ CFU/mL. A flask was added with 0.5 g powdered SilverSil. Another flask was added with 0.5 g of blank material (an ORMOSIL powder of identical composition but containing no silver). Finally, a third flask with no material added was used as control. All flasks were sterilized in autoclave at 121 °C. A sample (5 mL) of buffer phosphate suspension of the bacteria was added to each flask.

The resulting suspensions (solutions, in the case of no material added) were incubated at 37 °C for 1 hour under gentle shaking. After 1 hour, a small sample (900 μL) of each mixture was retrieved and transferred to an Eppendorf tube. The tube was centrifuged at 400 rpm for 30 s after which 100 μL of the upper liquid layer were transferred to a Petri dish agar plate for bacterial lawn. The plates were incubated at 37 °C for 24 hours after which bacteria were counted by the colony‐forming units (CFU) technique.

All SilverSil xerogels showed outstanding antibacterial activity against *S. aureus* (green bars in Figure 4), and *E. coli* (blue/yellow bars) bacteria. The 70 % methyl‐modified matrix had the highest activity against *Staphylococcus aureus*, hence it was selected as the optimal SilverSil antibacterial material.

**Figure 4:**
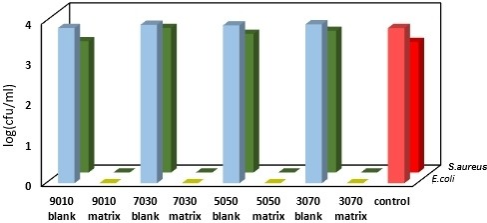
Antibacterial activity of different (10 %, 30 %, 50 % and 70 % methyl‐modified) SilverSil xerogels against *S. aureus* (green bars), and *E. coli* (blue bars). The activity of different blank matrices and of the control solution is also plotted (red bars).

In order to check whether the antibacterial activity of the SilverSil coatings was due to leached Ag nanoparticles, atom clusters or Ag ions, we measured the leaching of Ag in solution by washing the 70 : 30 SiverSil xerogel with water and different aqueous solutions in several consecutive washing cycles (Table 2). The silver concentration in the washing solutions was measured each time by inductively coupled plasma atomic emission spectroscopy (ICP‐AES) using a Spectro Arcos (Ametek, Berwyn, PA) instrument.

**Table 2:** Leaching of Ag in solution from the 70 : 30 SiverSil xerogel measured in different washing cycles via ICP‐AES.

Washing cycle	Ag concentration [ppm]	Amount of Ag leached [mmol]	Ag leaching [%]^a^
1	0.256	4.75 ⋅ 10^−5^	0.15
2	0.486	9.01 ⋅ 10^−5^	0.28
3	0.174	3.23 ⋅ 10^−5^	0.10
4	0.468	8.68 ⋅ 10^−5^	0.27
5	1.176	54.5 ⋅ 10^−5^	1.68
6	0.516	4.78 ⋅ 10^−5^	0.15
7	0.386	3.58 ⋅ 10^−5^	0.11

^a^Leaching percentage based on the total of 3.25×10^−2^ mmol silver encapsulated in the xerogel matrix.

The first four cycles (entries 1–4 in Table 2) were performed by immersing the SilverSil matrix in 20 mL water for 30 min, followed by filtration. In the two subsequent cycles (entries 5–6), the matrix was immersed in 50 mL and 10 mL of buffer solution for 30 min and for 3 days, respectively. Finally, in cycle 7 the matrix was immersed in 10 mL water for 24 h.

Results in Table 2 show that the amount of Ag leached in solution was extremely low, amounting to 88.5 ⋅ 10^−5^ mmol Ag after 7 washing cycles. Both the washing solutions and the washed SilverSil matrix were tested for antibacterial activity. Remarkably, regardless of modest leaching, *both* showed high antibacterial activity.

We ascribe the surprisingly high AB activity of the washing solutions to the lethal effect of the Ag^+^ present in solution in ultralow concentration, in a similar fashion to the 2 ppb Ag residual on textiles after prolonged washing inhibiting >99.9 % of *Escherichia coli* growth.^9^

The antibacterial activity of SilverSil is due to contact between the bacteria adsorbed and concentrated at the ORMOSIL outer surface, and the caged Ag nanoparticles. The accessibility of the ORMOSIL mesopores to polymeric molecules has been lately demonstrated with a structurally analogous ORMOSIL functionalized with an organocatalyst employed in the oxidation of cellulose.^19^ We remind that ceramic pot filters treated with colloidal silver are since long used for water sanitation in many rural areas in Africa, South East Asia and Latin America.^20^ Unfortunately, the ceramic silver‐impregnated pot filter is rapidly clogged by colloidal particles reducing the rate of safe water production.^21^

This work provides the proof of concept that SilverSil is a broad scope antibacterial sol‐gel material capable to quickly kill Gram‐positive and Gram‐negative bacteria. Since ORMOSIL alcogels rapidly and strongly bind virtually to any surface (including ceramic pots, but also metal, wood, cellulose or plastic objects),^22^ it will be enough to modify the sol‐gel synthetic route via procedures well known in the chemistry of silica‐based sol‐gel materials,^23^ to make SilverSil available as a paint with which to functionalize the surface of any object needing prolonged antibacterial protection.

Considering the pronounced physical and chemical stability of ORMOSILs,^24^ but also their low cost, ease of preparation (and application) and excellent health and environmental profile,^25^ these findings open the route to the widespread utilization of SilverSil coatings in life‐saving applications such as the ceramic pot filter, as well as in consumer products and across domestic, hospital, school, industrial and commercial environments.

## Conflict of interest

The authors declare no conflict of interest.

## References

[1] A. Todd , A. J. Worsley , R. J. Anderson , P. W. Groundwater , Pharm. J. 2009, 283, 359.

[2] S. T. Cole , Philos. Trans. R. Soc. London Ser. B 2014, 369, 20130430. 24821916 10.1098/rstb.2013.0430 PMC4024223

[3] G. G. F. Nascimento , J. Locatelli , P. C. Freitas , G. L. Silva , Braz. J. Microbiol. 2000, 31, 247.

[4] J. L. Clement , P. S. Jarrett , Met.-Based Drugs 1994, 1, 467. 18476264 10.1155/MBD.1994.467 PMC2364932

[5] N. Gugala , J. Lemire , K. Chatfield-Reed , Y. Yan , G. Chua , R. J. Turner , Genes 2018, 9, 344. 10.3390/genes9070344 PMC6071238 29986482

[6] V. Pareek , R. Gupta , J. Panwar , Mater. Sci. Eng. C 2018, 90, 739. 10.1016/j.msec.2018.04.093 29853145

[7] R. Ben-Knaz Wakshlak , R. Pedahzur , D. Avnir , Sci. Rep. 2015, 5, 9555. 25906433 10.1038/srep09555 PMC5386105

[8] B. Calderón-Jiménez , M. E. Johnson , R. A. Montoro Bustos , E. K. Murphy , M. R. Winchester , J. R. Vega Baudrit , Front. Chem. 2017, 5, 6. 28271059 10.3389/fchem.2017.00006 PMC5318410

[9] R. B. Reed , T. Zaikova , A. Barber , M. Simonich , R. Lankone , M. Marco , K. Hristovski , P. Herckes , L. Passantino , D. H. Fairbrother , R. Tanguay , J. F. Ranville , J. E. Hutchison , P. K. Westerhoff , Environ. Sci. Technol. 2016, 50, 4018. 26927927 10.1021/acs.est.5b06043

[10] W.-T. Li , H.-W. Chang , W.-C. Yang , C. Lo , L.-Y. Wang , V. F. Pang , M.-H. Chen , C.-R. Jeng , Sci. Rep. 2018, 8, 5593. 29618730 10.1038/s41598-018-23737-0 PMC5884781

[11] L. Li , G. Hartmann , M. Döblinger , M. Schuster , Environ. Sci. Technol. 2013, 47, 7317. 23750458 10.1021/es3041658

[12] S. S. Gupta , A. Baksi , P. Roy , D. Deb , T. Pradeep , ACS Sustainable Chem. Eng. 2017, 59, 8310.

[13] T. Nawaz , S. Sengupta , ACS Sustainable Chem. Eng. 2018, 61, 600.

[14] B. Akkopru-Akgun , C. Durucan , J. Sol-Gel Sci. Technol. 2007, 43, 227.

[15] E. Albert , P. A. Albouy , A. Ayral , P. Basa , G. Csik , N. Nagy , S. Roualdes , V. Rouessac , G. Safran , A. Suhajda , Z. Zolnai , Z. Horvolgyi , RSC Adv. 2015, 5, 59070.

[16] E. A. González , N. Leiva , N. Vejar , M. Sancy , M. Gulppi , M. I. Azócar , G. Gomez , L. Tamayo , X. Zhou , G. E. Thompson , M. A. Páez , J. Mater. Res. Technol. 2019, 8, 1809.

[17] R. Ciriminna , V. Pandarus , R. Delisi , A. Scurria , F. Giordano , M. P. Casaletto , F. Béland , M. Pagliaro , Chem. Cent. J. 2016, 10, 61. 27790286 10.1186/s13065-016-0208-6 PMC5062902

[18] D. Avnir , Acc. Chem. Res. 1995, 28, 328.

[19] S.-H. Jun , S.-G. Park , N.-G. Kang , Polymers 2019, 11, 1044. 10.3390/polym11061044 PMC6631351 31197111

[20] D. S. Lantagne, *Investigation of the Potters for Peace Colloidal Silver Impregnated Ceramic Filter, Report 2: Field Investigations*, Alethia Environmental, Allston MA: 2001.

[21] D. van Halem , H. van der Laan , S. G. J. Heijman , J. C. van Dijk , G. L. Amy , Phys. Chem. Earth 2009, 34, 36.

[22] M. Pagliaro , R. Ciriminna , G. Palmisano , J. Mater. Chem. 2009, 19, 3116.

[23] M. Pagliaro, *Silica-Based Materials for Advanced Chemical Applications*, RSC Publishing, Cambridge: 2009.

[24] M. Pagliaro , R. Ciriminna , M. Wong Chi Man , S. Campestrini , J. Phys. Chem. B 2006, 110, 1976. 16471772 10.1021/jp055697v

[25] D. Kumar , I. Mutreja , P. K. Keshvan , M. Bath , A. K. Dinda , S. Mitra , J. Pharm. Sci. 2015, 104, 3943. 26295279 10.1002/jps.24614

